# Facts Regarding Succinimide of Mercury

**Published:** 1916-06

**Authors:** Geo. H. Reed

**Affiliations:** Dental Surgeon U. S. N.


### FURTHER FACTS REGARDING SUCCINIMIDE OF MERCURY AS A CURE FOR PYORRHEA.

BY DR. GEO. H. REED, A.A., DENTAL SURGEON U. S. N.

Since publishing in *Dental Items of Interest*, of April, 1914, an article embodying the results of six months' experimentation with succinimide of mercury as a remedy for pyorrhea alveolaris, I have been deluged with communications from Maine to California requesting additional data on the technique of the drug's administration; where it could be procured; its systemic effects; as to whether patients experienced any ill effects as a result of this treatment or not; whether any variations from the published results of the twenty odd cases which the article in question recorded had been observed in subsequent cases, and whether or not these alleged cures had remained cured. I have also been requested to publish for the information of those who are interested, the results of later injections of this drug and have been urged by Dr. Barton L. Wright, U. S. N., who first called the attention of the medical profession to the value of succinimide of mercury in the treatment of tuberculosis, arthritis and other kindred diseases, including pyorrhea, to make some statement of my opinion as to the curative value of this remedy in pyorrheal cases, based on its almost daily use in this office for over a year and four months.

There is only this to say. In every case I have completed and subsequently examined, there has been none in which I could detect the slightest trace of pyorrhea. The patients whom I have treated are universal in their approval of the treatment they have received, and some exceedingly garrulous in their satisfaction.

In two instances I have been obliged to extract. One tooth in each case. These were due to the denuded condition of the roots caused by the inroads of the infection, the deposition of salivary calculus, etc., before curative measures were undertaken.

### Variation in Effects.

One variation in the effects of the mercurial injection is marked. This is the rate of absorption of the drug into the system. Just why this variation occurs is not clear, being due probably to the amount of resistance encountered in the tissues of the individual; but the fact that this resistance is greater in some than in others, has no effect on the curative value of the drug, merely postponing its effects and delaying the cure.

The female clientele of this office is not large, but in the few cases I have been called upon to treat, the rate of absorption is considerably slower than in the male sex. One young woman in particular, whose treatment was instituted last September, is yet (this is being written in January) carrying about a lump the size of a small pea at the seat of the last injection made in November, while the pyorrhea is entirely cleared up. This case has been of more than ordinary interest owing to the length of time it has taken to accomplish the cure, due entirely to the delay in absorption of the mercury, the relatively small quantity used, and the virulence of the infection. This patient had also received three months before being sent to me for treatment, six subcutaneous injections of emetine. A matter of further interest lies in the fact that the patient also complained of a feeling of lassitude and what might be described as a general lessening of systemic tone immediately after, and for several days subsequent to the injection of the drug.

Other than this no ill effects have been noticed. The majority of patients treated at this office are in excellent physical health, and aside from a certain soreness and uncomfortable feeling at the point of injection, no ill effects have been reported or observed.

In relation to a recurrence of the infection, patients in this office are under military discipline and there are few failures in keeping appointments. I have made it a point to send for and examine cases treated as often as possible when these cases were in port, and have thus far never found a recurrence nor registered a complaint.

The technique of administration remains the same in effect as contained in the article published last April, and over fifty patients have been given injections varying from one to four in each case, and of a size in dosage, measured by the one-fifth grain tablet, as the requirements of their individual cases seemed to indicate.—*Items of Interest.*

